# Occurrence of *Toxoplasma gondii* in cattle and sheep in Akmola and Kostanay regions of Kazakhstan

**DOI:** 10.14202/vetworld.2024.2944-2949

**Published:** 2024-12-26

**Authors:** Kanat Tursunov, Laura Tokhtarova, Zhansaya Adish, Raikhan Mustafina

**Affiliations:** 1 Laboratory of Immunochemistry and Immunobiotechnology, National Center for Biotechnology, 010000, Astana, Kazakhstan; 2 Department of Natural Sciences, L. N. Gumilyov Eurasian National University, 010008, Astana, Kazakhstan; 3 Department of Veterinary Sanitation, Faculty of Veterinary and Animal Husbandry Technology, S. Seifullin Kazakh Agrotechnical Research University, 010000, Astana, Kazakhstan

**Keywords:** antibodies, cattle, enzyme-linked immunosorbent assay, sheep, surface antigen 1, *Toxoplasma gondii*

## Abstract

**Background and Aim:**

*Toxoplasma gondii* is an intracellular protozoan and a major foodborne pathogen worldwide. Nearly, all warm-blooded animals are susceptible to toxoplasmosis, with raw and undercooked meat and animal products serving as the primary transmission routes. To date, the distribution of *T. gondii* among farm animals in Kazakhstan has been inadequately studied. This study aimed to determine the occurrence of antibodies against *T. gondii* in cattle and sheep in the Akmola and Kostanay regions.

**Materials and Methods:**

Blood samples were randomly collected from 437 cattle and 397 sheep from two regions of Kazakhstan: Akmola and Kostanay. A commercial enzyme-linked immunosorbent assay based on the native protein *Toxoplasma* surface antigen 1 was used for serological analysis.

**Results:**

The occurrences of *T. gondii* were 8.0% and 3.8% among cattle and 42.1% and 19.0% among sheep in the Akmola and Kostanay regions, respectively. Antibodies against *T. gondii* were detected in all study areas. The greatest frequency of seropositive reactions in cattle was observed in the Arshaly region (9.0%), whereas the least frequent was observed in Arkalyk (3.3%). The highest occurrence of seropositive reactions among sheep was found in the Zerenda region (54.5%), whereas the lowest was found in the Auliekol region (15.6%).

**Conclusion:**

The obtained results confirmed the circulation of the *T. gondii* pathogen among cattle and sheep in the investigated regions. These findings provide insight into the current distribution of this zoonotic parasite among farm animals in Kazakhstan.

## Introduction

Toxoplasmosis, caused by the protozoan parasite *Toxoplasma gondii*, remains a significant concern in veterinary and public health because of its widespread prevalence among warm-blooded animals [1]. It affects approximately one-third of the global population, posing significant economic and social concerns, particularly concerning public health and livestock production [2, 3]. Cats are the primary reservoirs of the parasite, excreting many *T. gondii* oocysts through their feces. Once sporulated, these oocysts can remain infectious in water and soil for extended periods, subsequently infecting humans and animals [4, 5].

Human infection typically occurs through consumption of raw or undercooked meat from infected animals or through exposure to oocyst-contaminated food and water [6–8]. Although the disease is often asymptomatic, it can manifest as influenza-like symptoms or other non-specific clinical signs [9]. In immunocompromised individuals, however, toxoplasmosis can become severe and potentially life-threatening [10]. Because there is a chance of vertical transmission from mother to fetus, pregnant women are particularly at risk. This can result in serious complications, such as neurological or ocular disorders, hydrocephalus, mental retardation, seizures, intrauterine mortality, and abortion [11–13]. In farm animals, *T. gondii* infection may lead to abortion, fetal mummification or maceration, intrauterine embryonic death, stillbirth, or postnatal mortality, all of which jeopardize livestock production. Surviving offspring is often weak and susceptible to other diseases [14]. The productivity of livestock is closely tied to reproductive efficiency, and high fetal mortality rates caused by various pathogens, including protozoa [15], bacteria [16], and viruses [17] are significant sources of economic losses in the agricultural sector, particularly on ruminant farms.

To date, the prevalence of toxoplasmosis in Kazakhstan has remained inadequately studied. In 2009, serological testing of rural populations in East Kazakhstan revealed that 16.1% (504/3,126) of individuals tested positive for toxoplasmosis [18]. However, data on the spread of toxoplasmosis among cattle and sheep in Kazakhstan are sparse, despite these animals being a major source of meat in the country and a potential vector for the transmission of *T. gondii* to humans [2].

Thus, this study aimed to determine the occurrence of antibodies against *T. gondii* in cattle and sheep in the Akmola and Kostanay regions of Kazakhstan.

## Materials and Methods

### Ethical approval

This study was approved by the Ethics Committee of the National Center for Biotechnology, Astana, Kazakhstan (Protocol #11, 16/11/2022).

### Study period and location

The study was conducted from October 2023 to May 2024 in the Akmola and Kostanay regions of Kazakhstan. Laboratory analyses were performed at the National Center for Biotechnology in Astana, Kazakhstan.

### Serum samples

**Figure-1:**
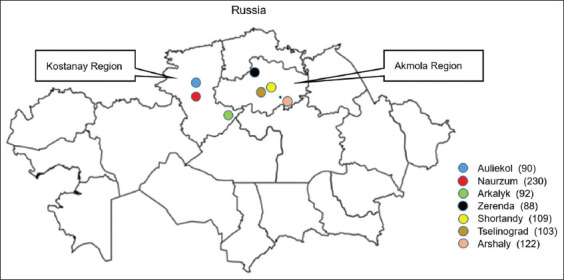
Location of sampling for the study of the occurrence of *Toxoplasma* *gondii* infection in cattle and sheep in the Akmola and Kostanay regions [Source: www.supercoloring.com/ru/raskraski/konturnaya-karta-kazahstana-s-regionami? version=print].

In total, 834 serum samples were randomly collected from farm animals, consisting of 437 samples from cattle and 397 samples from sheep. Serum samples from cattle (n = 301) and sheep (n = 111) were collected from different districts of the Akmola and Kostanay regions (Figure-1). In addition, 136 bovine serum samples and 286 sheep serum samples were obtained from previous brucellosis-related experiments conducted from 2020 to 2023. Blood serum samples were collected from animals on small farms and private households with no specific criteria applied to different farms. The ages of the cattle ranged from 2 to 6 years and the sheep ranged from 1 to 5 years. Blood was drawn from the jugular vein into vacutainer using a clot activator. The tubes were then centrifuged at 200× *g* for 10 min, and the serum was separated and stored at −20°C until further analysis.

### Enzyme-linked immunosorbent assay (ELISA)

ELISA was performed using a commercial ELISA kit (ID Vet, Montpellier, France) to detect *T. gondii* immunoglobulin G (IgG) antibodies. Serum samples were diluted 1:10 in advance on a separate plate and then transferred to the working plate using a multichannel dispenser. After 45 min of incubation at 25°C, the plate was washed, and 100 μL of 1× conjugate solution was added to each well. The plate was then incubated for 30 min at 25°C, followed by another wash. To develop the reaction, 100 μL of substrate solution was added to each well, and the plate was incubated for 15 min at 25°C in a dark place. The optical density (OD) was measured at 450 nm. The test was deemed valid if the average OD of the positive control was >0.35, and the ratio of the average OD values of the positive control to the negative control was >3. Samples with S/P % readings >50% were considered positive, those below 40% were classified as negative, and samples with readings between 40% and 50% were deemed doubtful.

### Statistical analysis

The occurrence of *T. gondii* serum antibodies was calculated as the proportion of seropositive samples among the total number of tested samples. The mean OD values were compared using paired Student’s *t*-test, with differences considered statistically significant at p < 0.05.

## Results

### Occurrence of *T. gondii* serum antibodies in cattle and sheep

**Figure-2:**
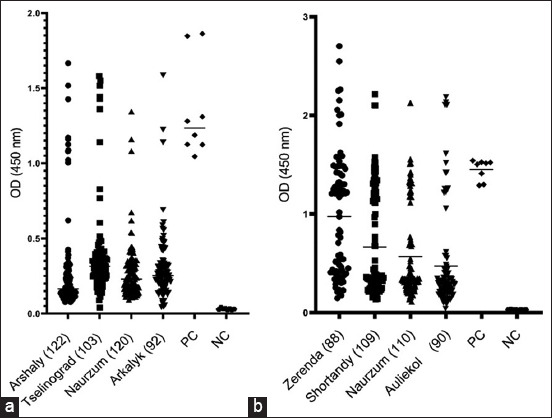
Occurrence of *Toxoplasma gondii* in (a) cattle and (b) sheep from different districts by ELISA. Each symbol represents a separate serum sample. PC=Positive control, NC=Negative control, ELISA=Enzyme-linked immunosorbent assay.

In total, 834 serum samples from cattle and sheep across various regions of Kazakhstan were tested for specific IgG antibodies against *T. gondii* using the ELISA method (Figure-2). The average OD values of the positive control significantly exceeded the value of 0.35, and the ratio of the average OD values of the positive control to the negative control was >3, which demonstrates the reliability of the test. Analysis of the OD of cattle sera (Figure-2a) in a 1:10 dilution showed that the average titer of negative samples varied from 0.1 to 0.5. The OD values of positive samples were within the limits of the positive control. The average OD value of sheep sera (Figure-2b) was higher than that of cattle and varied within 0.1–1.0 for negative samples and >1.1 for positive samples. At the same time, some samples had OD values significantly higher than those of the positive control. The cutoff value for each test was calculated using the manufacturer’s formula depending on the OD values of the control and test samples.

The overall occurrence rate of all animals was 17.6%. In the cattle, the average occurrence was 5.9% (Table-1). The Akmola region exhibited a higher occurrence rate than Kostanay (8.0% and 3.8%, respectively). In the Akmola region, the occurrence rates were 9.0% in Arshaly and 6.8% in Tselinograd (p < 0.05). In the Kostanay region, the occurrence rates were 4.2% in Naurzum and 3.3% in Arkalyk (p > 0.05).

**Table-1:** Occurrence of *Toxoplasma gondii* in cattle in different districts of Kazakhstan.

Region	District	Number of tested samples	Positive, n (%)	Negative, n (%)	Doubtful, n (%)	p-value
Akmola	Arshaly	122	11 (9.0)	111 (91.0)	-	0.046
	Tselinograd	103	7 (6.8)	94 (91.3)	2 (1.9)	
Kostanay	Naurzum	120	5 (4.2)	115 (95.8)	-	0.081
	Arkalyk	92	3 (3.3)	89 (96.7)	-	
Total		437	26 (5.9)	409 (93.6)	2 (0.5)	

Interestingly, the overall occurrence rate among sheep was significantly higher at 30.5%, more than 5 times the rates observed in cattle (Table-2). Similar to the findings for cattle, occurrence among sheep was higher in the Akmola region than in Kostanay, with rates of 42.1% and 19.0%, respectively. In Akmola, the highest occurrence rates were recorded in the Zerenda (54.5%) and Shortandy (32.1%) districts (p > 0.05). In the Kostanay region, the occurrence rates were 21.8% in Naurzum and 15.6% in Auliekol (p < 0.05).

**Table-2:** Occurrence of *Toxoplasma gondii* in sheep in different districts of Kazakhstan.

Region	District	Number of tested samples	Positive, n (%)	Negative, n (%)	Doubtful, n (%)	p-value
Akmola	Zerenda	88	48 (54.5)	40 (45.5)	-	0.185
	Shortandy	109	35 (32.1)	70 (64.2)	4 (3.7)	
Kostanay	Naurzum	110	24 (21.8)	78 (70.9)	8 (7.3)	0.039
	Auliekol	90	14 (15.6)	74 (82.2)	2 (2.2)	
Total		397	121 (30.5)	262 (66.0)	14 (3.5)	

## Discussion

Animal husbandry is a key industry in Kazakhstan. According to the Bureau of National Statistics of Kazakhstan, the current livestock population includes 9.6 million cattle and 26.5 million small ruminants. Raw meat, along with meat and dairy products from these animals, has been identified as a potential cause of human toxoplasmosis [19]. However, there is a lack of sufficient information on the spread of toxoplasmosis among farm animals in Kazakhstan, making serological monitoring of livestock crucial for veterinary and public health.

Timely and accurate diagnosis of toxoplasmosis is essential for effective disease control. In this study, we used a commercial ELISA kit (ID Vet). This ELISA is based on the native protein P30 (surface antigen 1 [SAG1]), the most immunodominant protein of *T. gondii* [20]. SAG1 is highly conserved among coccidian parasites, such as *Neospora caninum*, and may cause cross-reactivity. For example, serological cross-reactivity between *T. gondii* and *N. caninum* was observed using ELISA. However, cross-reactivity was significantly influenced by serum sample dilution, with higher dilutions reducing cross-reactivity [21, 22]. The ELISA kit is widely used for the serological monitoring of toxoplasmosis in both farm and domestic animals. For example, Mohamed-Cherif *et al*. [23] conducted serological monitoring of cattle, sheep, and goats using an ELISA kit and reported seroprevalence rates of 28.7%, 25.6%, and 11.9%, respectively. Pablos-Tanarro *et al*. [24] compared this ELISA with the direct agglutination test in a serological study of 2492 sows, finding toxoplasmosis prevalence rates of 5.8% and 8.9%, respectively. Similarly, Dahmane *et al*. [25] estimated *T. gondii* seroprevalence in goats using both ELISA and latex agglutination tests, finding seroprevalence rates of 71.73% and 63.58%, respectively, in 184 serum samples from goats in northeastern Algeria. Xia *et al*. [26] used this ELISA to test 1521 domestic and wild cats, reporting seroprevalence rates of 4.2% in urban cats and 20.9% in stray cats, with regional prevalence ranging from 0.9% to 13%.

In this study, we determined the occurrence of antibodies against *T. gondii* among cattle and sheep in the Akmola and Kostanay regions of Kazakhstan. Antibodies against *T. gondii* were detected in all study areas. Among the 437 cattle serum samples analyzed, the overall occurrence rate was 5.9%, a relatively low rate. Some studies suggest that cattle are less susceptible to toxoplasmosis than other ruminants [27, 28]. However, previous studies have indicated considerable variability in the occurrence of toxoplasmosis among cattle worldwide. For example, the occurrence rate observed in this study is consistent with results from northern Europe (7%) [29], Algeria (4.4%) [30], and Brazil (5.3%) [31]. Higher rates have been reported in studies conducted in Italy (10.2%) [32], Mongolia (18.7%) [33], and northern China (31.3%) [34].

In contrast, in our study, the occurrence rate in sheep was significantly higher, with an average of 30.5%. The antibodies against *T. gondii* ranged from 15.6% in the Auliekol district of the Kostanay region to 54.5% in the Zerenda district of the Akmola region. Specifically, occurrence rate in the Akmola and Kostanay regions was 8.0% and 3.8% among cattle and 42.1% and 19.0% among sheep, respectively. These results highlight variations not only between countries but also within regions of the same country. We assume that various factors, such as climate, sample size, animal breed, housing system, and level of disease prevention and control, could have influenced this difference [35]. In addition, the distance between the Akmola and Kostanay regions (over 400 km) should be considered. Therefore, we plan to conduct serological studies covering more regions and livestock in the future.

The results indicate a potentially high risk of toxoplasmosis infection among the population of the mentioned regions. As noted by Torgerson *et al*. [18], the rate of seropositivity against *T. gondii* in rural populations was 16%. This is likely related to the fact that livestock farming is the primary source of income in rural areas, meaning that a large number of people have daily contact with animals. In addition, people whose work is closely associated with animals, raw meat, or animal offal are also at risk. These include veterinarians, livestock handlers, slaughterhouse workers, and farmers. Serological studies have shown that this group has an anti-*T. gondii* IgG seropositivity rate ranging from 46% to 72.8%, which is significantly higher than that in the general population [36–38].

The main limitations of the study include the relatively small number of farms sampled and the lack of data on the age and sex of the livestock. Further serological studies are needed to improve our understanding of the epidemiology of toxoplasmosis in ruminants in Kazakhstan. Our study represents a preliminary analysis, laying the groundwork for more extensive research. This will ultimately help assess the risk of human infection associated with the consumption of contaminated meat.

## Conclusion

Our study confirmed the presence of *T. gondii* in cattle and sheep in all the studied areas. Overall, 147 (17.6%) of the 834 animals tested were seropositive, with 26 (5.9%) of cattle and 147 (30.5%) of sheep showing evidence of infection. These results suggest that toxoplasmosis poses a significant threat to both farm animals and humans in the study area. To better evaluate the potential public health risk, larger-scale serological studies involving livestock from other regions in Kazakhstan are recommended.

## Authors’ Contributions

KT and RM: Designed and supervised the study and drafted and revised the manuscript. LT and ZA: Conducted the experiments and analyzed the data. All authors have read and approved the final manuscript.
